# Supplementary material for: A Physics-Informed Neural Network approach for compartmental epidemiological models
Source: PLoS Comput Biol. 2024 Sep 5;20(9):e1012387. doi: 10.1371/journal.pcbi.1012387 (PMC11407682; doi:10.1371/journal.pcbi.1012387)
Supplement: S1 Text — Appendix A. Neural Network architectures. Appendix B. Full SIR model with large data errors. Appendix C. Forecasting using the joint PINN approach. Appendix D. Comparison with the renewal equation. (PDF) [file pcbi.1012387.s001.pdf]

Supporting information for

A Physics-Informed Neural Network approach for compartmental epidemiological models

Caterina Millevoi[1*], Damiano Pasetto[2], Massimiliano Ferronato[1],

1 Department of Civil, Environmental and Architectural Engineering, University of Padova, via Marzolo 9, Padova, Italy
2 Department of Environmental Sciences, Informatics and Statistics, Ca' Foscari University of Venice, Via Torino 155, Venezia Mestre, Italy

* caterina.millevoi@unipd.it

Appendix A. Neural Network architectures.

We perform a comparison between several NN architectures with the aim of searching for a balance between small approximation errors and affordable training times of the PINN method. The analysis is carried out in the setting of Case 1, with constant β_0 parameter. We let the number of layers and neurons respectively vary in {4,10} and {5,25,50,100}, for a total of 8 different architectures. The other training hyperparameters are fixed as described in Section 2.1. For each architecture we train the joint PINN-based model for 5000 epochs.

The relative errors on the trained state variables and the parameter β (36) are plotted in Fig A for each architecture. Fig B shows the sum of the relative errors, the training time, and the number of NN parameters in each architecture.

The results with 10 layers show that the error increases as the number of neurons grows. Such behavior should not be surprising, since it is strictly correlated with the increase of the NN depth and the total number of parameters (see Fig Bc). The training time and the number of training points needed to train the NNs are larger for deeper NNs, since they are characterized by a larger number of unknown parameters. The analysis shows that, if we consider accuracy and training time, the best choices are either a NN with 4 layers and 25 neurons each, or one with 4 layers and 50 neurons. Since the goal is to build a general model for all the applications, for $\hat{S}_s$ and $\hat{I}_s$ we choose the number of layers and neurons equal to 4 and 50, respectively. This entails model's higher expressiveness at a limited training computational cost. The analysis is consistent with other results obtained in literature [1], which underline that in PINNs, higher complexity models often result in larger errors. In fact, PINN errors account for three inseparable components: the approximation error, the generalization error, and the optimization error, each influenced by various factors like the residual collocation points and the loss function complexity. Consequently, PINN training on noisy datasets typically shows that more complex NNs can be less effective, achieving minimum global error at an optimal trade-off architecture.

A further NN $\hat{\beta}$ is needed to approximate the time-dependent transmission rate (Case 2). In this case the sensitivity analysis is performed only on this NN, while the NNs for the state variables are set to have 4 layers and 50 neurons. Fig C shows that some spikes arise when approximating β with a NN made of only 4 layers and 50

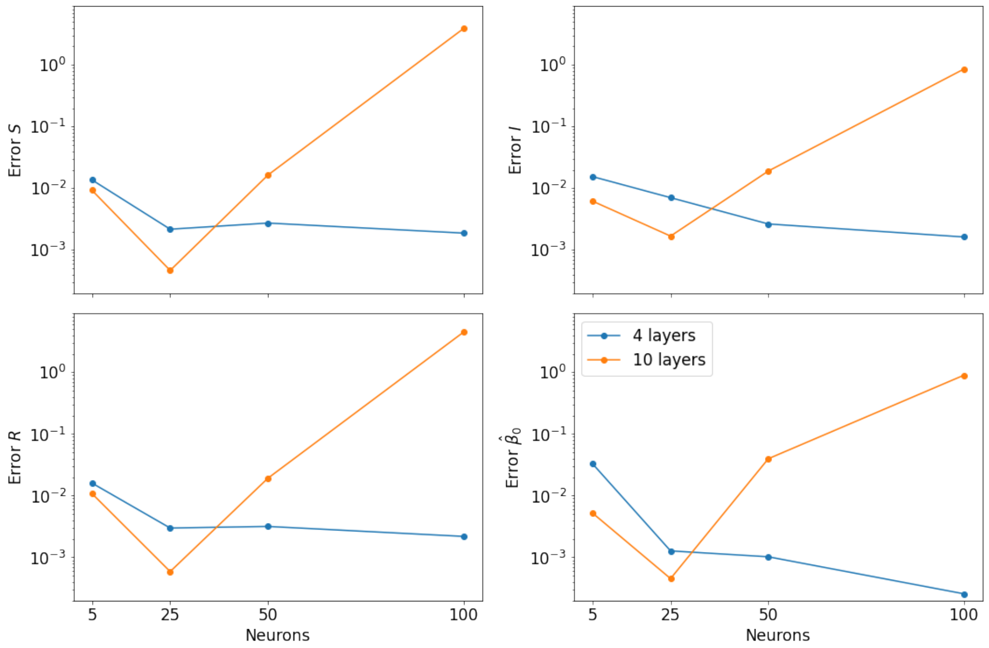

Fig A. Errors on the state variables associated to the different NN architectures in Case 1.

neurons. This issue is not present when we double the number of neurons. For this reason, we select 4 layers and 100 neurons for the unknown time-dependent parameters.

Weights of the loss function.

As described in the main article, the PINN loss function takes into account the contribution of different error terms. These terms are typically weighted, in order to obtain similar order of magnitudes. The SciANN package has the possibility to calibrate the weights during the PINN training by using an NTK adaptive update. Table A shows the improvement of the errors obtained in Case 1 by using the NTK or not (the different terms of the loss function are summed with all weights equal to 1).

	NTK calibration	No adaptive weights
Training time [s]	2223	1914
Error S	2.763×10^{-3}	4.507×10^{-2}
Error I	3.846×10^{-3}	5.899×10^{-2}
Error R	3.258×10^{-3}	5.202×10^{-2}
Error β	5.086×10^{-3}	1.071×10^{-1}
PINN $\hat{\beta}$	0.59738	0.53579

Table A. Impact of the NTK calibration of the loss-term weights during the training of the joint PINN-based model in Case 1.

The results show that the NTK calibration of the weights during the training entails a considerable decrease of the model errors at a limited cost in terms of training duration.

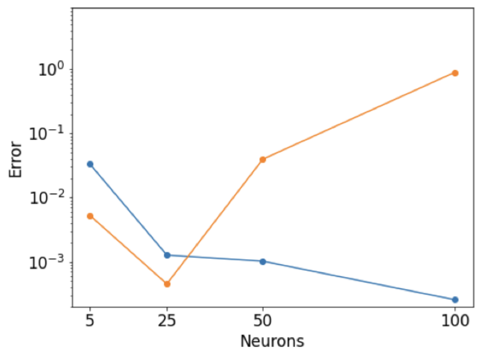

(a)

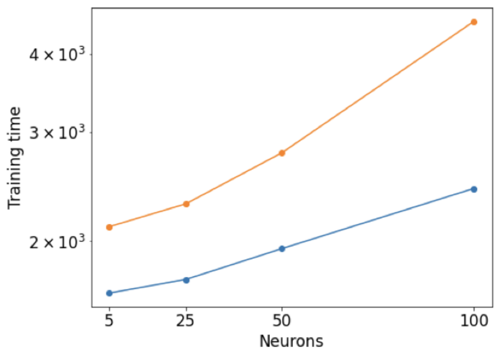

(b)

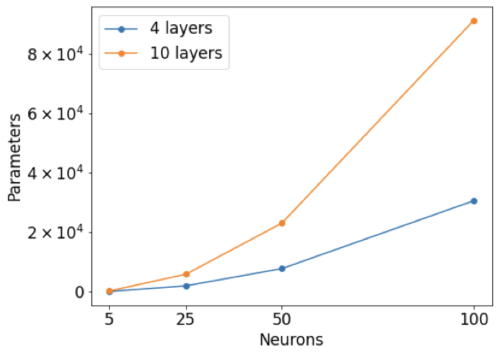

(c)

Fig B. Total error (a), training time (b), and number of parameters (c) associated to different NN architectures in Case 1.

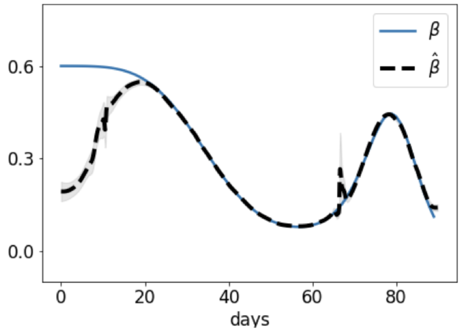

(a)

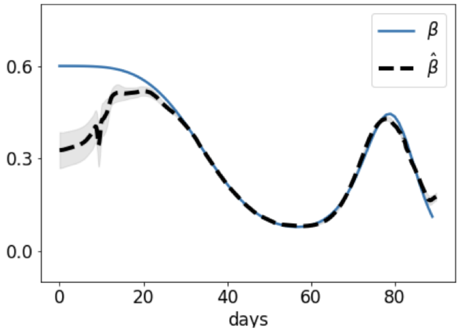

(b)

Fig C. Comparison between reference time-dependent transmission rate β and its PINN estimation in Case 2, when using the following architectures for $\hat{\beta}$: (a) 4 layers with 50 neurons and (b) 4 layers with 100 neurons.

Appendix B. Full SIR model with large data errors.

The full SIR model is here applied to Case 4, with synthetic transmission rate and large data errors for the infectious compartmental class. The results highlight a clear difficulty of the model to process data affected by large noise both using the joint and split approach (Fig. D), that corroborates the relevance of the proposed reduced approach in real-world applications.

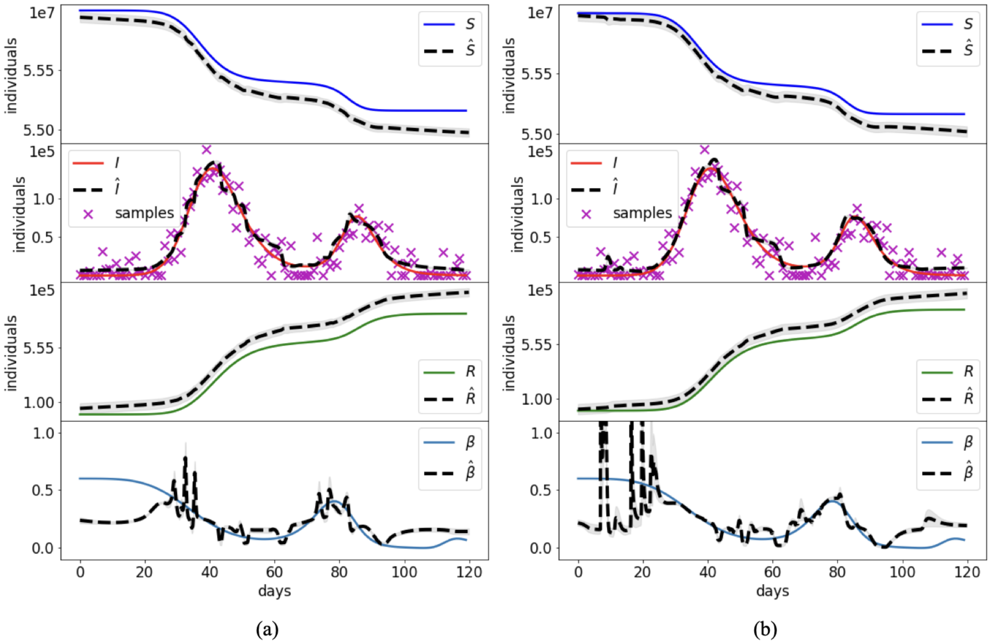

Fig D. Case 4: full SIR model with large data errors. Comparison between the reference solution of the SIR model and the PINN approximations with the joint (a) and split (b) approach. Grey bands provide the confidence interval for one standard deviation.

Appendix C. Forecasting using the joint PINN approach.

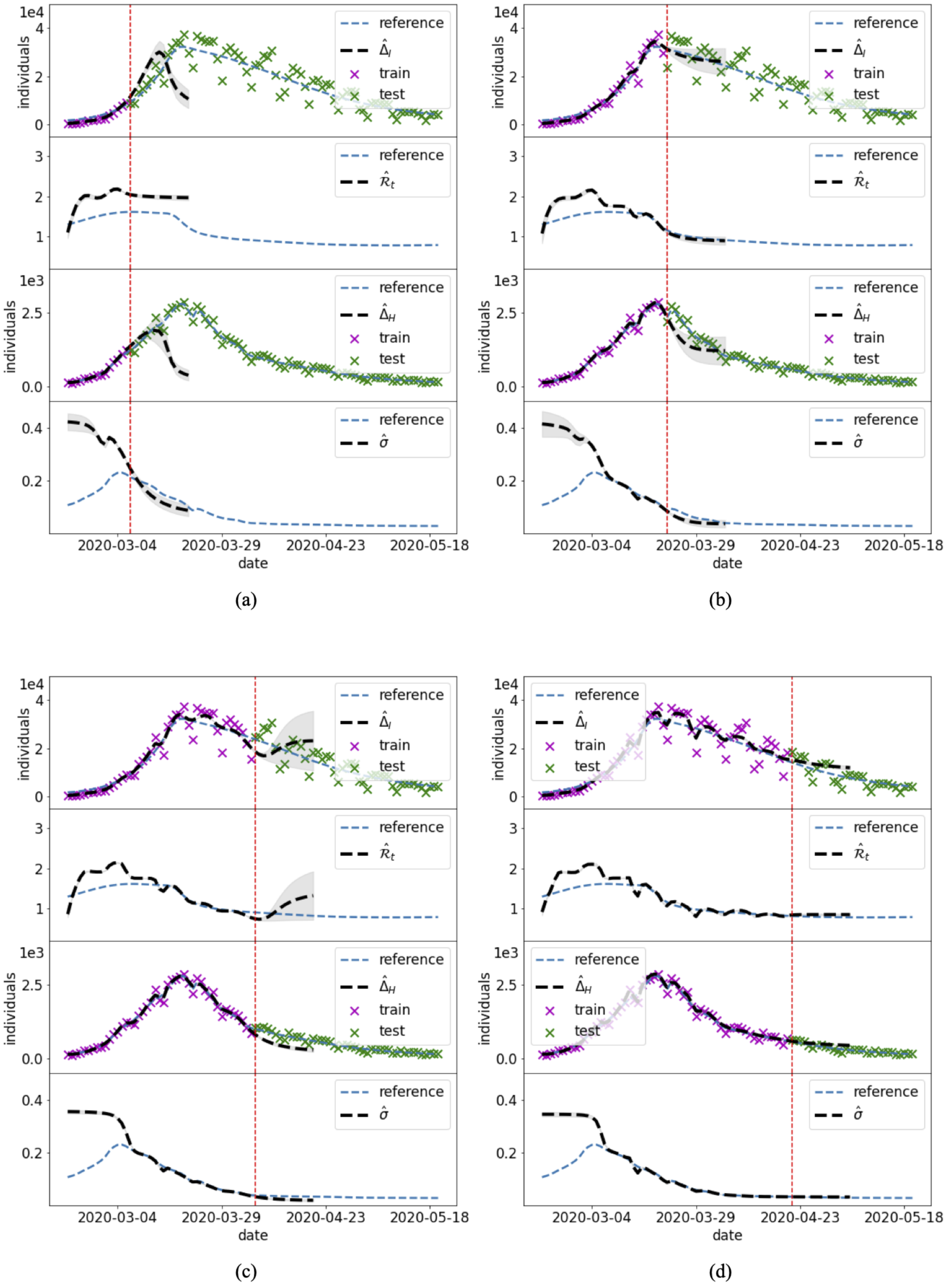

Fig E. Forecasting using the joint PINN approach. PINN predictions of the Italian COVID-19 evolution using the joint method on subsequent training windows: (a) 0-15 days, (b) 0-30 days, (c) 0-45 days, (d) 0-60 days (same procedure as described in Section 2.2.2). The blue dashed lines are the output of the joint approach computed in Case 7 (Fig 11a) and are plotted as reference result. If compared with the joint approach, the split training outperforms it in terms of training time and is less prone to overfitting.

Appendix D. Comparison with the renewal equation.

A traditional approach to infer $\mathcal{R}_t$ is based on Bayesian inference applied to the renewal equation. We synthetically present this approach here for a comparison with the PINN-based method. Please refer to [2, 3] for more details. The main idea is that the mean number of infected individuals on a day t, $\bar{\Delta}_I(t)$, should be proportional to the weighted sum of the past recorded daily infections Δ_I:

$$\bar{\Delta}_I(\mathcal{R}_t) = \mathcal{R}_t \sum_{j=t-k}^{t-1} \phi(j)\Delta_I(t-j) \tag{SI.1}$$

where the weight function ϕ is the probability density function (pdf) of the generation times, truncated after k days. Then, $\phi(j)$ represents the probability density that an infected individual can transmit the disease after j days of infection. The data collected on day t, $\Delta_I(t)$, is subject to counting errors that follow an observation error pdf with expected value $\bar{\Delta}_I(\mathcal{R}_t)$, in the following indicated as $p_I(\Delta_I \mid \bar{\Delta}_I(\mathcal{R}_t))$. The posterior probability of $\mathcal{R}_t$ can be sampled using, for example, a Markov Chain Monte Carlo on the likelihood

$$\mathcal{L}(\mathcal{R}_t \mid \Delta_I(t)) = p_I(\Delta_I \mid \bar{\Delta}_I(\mathcal{R}_t))p_0(\mathcal{R}_t) \tag{SI.2}$$

where $p_0(\mathcal{R}_t)$ is the prior probability distribution of $\mathcal{R}_t$. To avoid strong oscillations on the inferred $\mathcal{R}_t$ values, [2] suggests considering it constant over a temporal window of length l. Assuming that the daily counting errors are independent, the likelihood function for $\mathcal{R}_t$ takes into account the product of the observation errors pdfs on each day of the window.

We compared the posterior pdf obtained using this approach with the split PINN results in Case 3 (synthetic and accurate data, based on the $\mathcal{R}_t$ of the Italian COVID-19 outbreak) and Case 4 (synthetic data subject to large errors). In both cases the generation times ϕ have an exponential distribution with mean value $\delta^{-1} = 0.2\ d^{-1}$ (which corresponds to the distribution of the generation times in the SIR model).

In accordance to the generation of the measurements in the selected scenarios, in Case 3 the observation error pdf p_I has a Poisson distribution with mean $\bar{\Delta}_I(\mathcal{R}_t)$. In Case 4, p_I has a Gaussian distribution with mean $\bar{\Delta}_I(\mathcal{R}_t)$ and standard deviation equal to 40% of the deviation of the data.

It is important to note that the PINN approach simultaneously considers all the data in the simulation to infer the temporal changes in $\mathcal{R}_t$. The renewal equation, instead, independently estimates $\mathcal{R}_t$ on short temporal windows, typically based on past data. For example, using a window of 5 days, the estimate on day t is obtained by considering $\mathcal{R}_t$ as constant on days $t-4, \dots, t-1, t$, which might cause a delay in the $\mathcal{R}_t$ changes. To make the comparison more fair, we modified the method by averaging the values of $\mathcal{R}_t$ on a centered windows of length 5 days (2 days in the past and 2 in the future).

The results are presented in Figs F and G for Cases 3 and 4, respectively.

The comparison shows similar results, in both cases. In Case 3 the renewal equation clearly outperforms the $\mathcal{R}_t$ estimation of PINNs at the beginning of the epidemic. The adaption of the training approach described in Section 2.2.2 of the manuscript where PINN is sequentially applied in larger time windows, allows to improve the $\mathcal{R}_t$ estimates at the beginning of the simulation (see Fig F). At larger times, the PINN estimation better agrees with the reference values in both Cases 3 and 4. The main advantage of the approach based on the renewal equation is evident in Case 4, where the data is subject to larger errors. In fact, the method provides an estimation of the confidence interval which is intrinsic to this Bayesian approach.

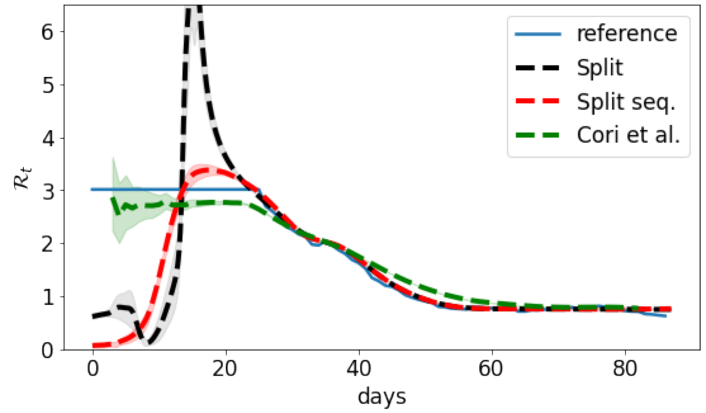

Fig F. Comparison on Case 3. Comparison between the $\mathcal{R}_t$ estimates obtained using the split PINN approach as in 2.1.2) (the black dashed line is the mean and the grey shaded area is the 95% confidence interval among 10 trainings) and using the renewal equation of [2] (the green dashed line is the mean and green shaded area is the 95% confidence interval of 9000 samples). The blue line is the reference value as in Case 3. The results of using the split PINN approach using a sequential training are in red.

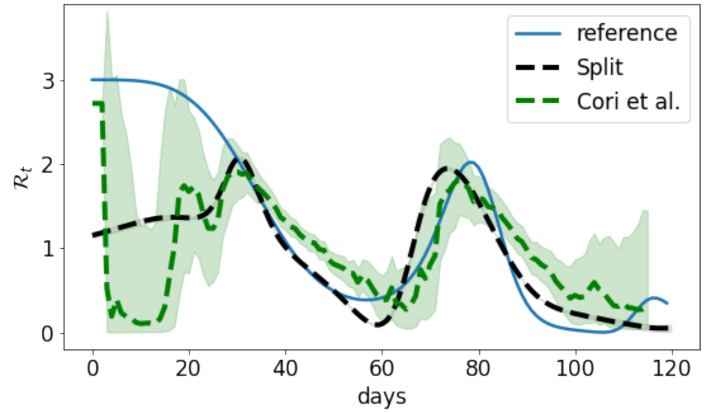

Fig G. Comparison on Case 4. As in Fig F for Case 4 (larger data errors).

References

1. Lu L, Meng X, Mao Z, Karniadakis G E. DeepXDE: A Deep Learning Library for Solving Differential Equations. SIAM Review. 2021;63:208-228. doi:10.1137/19M1274067.

2. Cori A, Ferguson NM, Fraser C, Cauchemez S. A new framework and software to estimate time-varying reproduction numbers during epidemics. Am J Epidemiol. 2013;178(9):1505–1512. doi:10.1093/aje/kwt133.

3. Pasetto D, Lemaitre JC, Bertuzzo E, Gatto M, Rinaldo A. Range of reproduction number estimates for COVID-19 spread. Biochemical and Biophysical Research Communications. 2021;538:253–258. doi:10.1016/j.bbrc.2020.12.003.
