# Supplementary material for: A Physics-Informed Neural Network approach for compartmental epidemiological models
Source: PLoS Comput Biol. 2024 Sep 5;20(9):e1012387. doi: 10.1371/journal.pcbi.1012387 (PMC11407682; doi:10.1371/journal.pcbi.1012387)
Supplement: Attachment — Submitted filename: review_PLOS.pdf [file pcbi.1012387.s002.pdf]

Review PCOMPBIOL-D-23-02037

February 2024

Title

A Physics-Informed Neural Network approach for compartmental epidemiological models.

Summary of paper

The paper introduces an innovative approach to address inverse problems related to epidemiological data and utilizing PINNs. Specifically, two simple innovative modifications, named the reduced and split approaches, are introduced and compared in a comprehensive campaign of numerical results involving both synthetic and realistic scenarios.

Strengths

- The authors have shown a clear understanding of the existing state-of-the-art about Machine Learning techniques for solving Inverse Problems and in particular the application to the epidemiological context;
- The proposed methods introduce little modifications adapted to the epidemiological framework to the well-established framework of PINNs.

Major Comments

- I believe there is a lack of explicit justification for employing Neural Networks with large dimensions, as indicated in lines 369-373, particularly regarding their depth and width. Could this choice be somehow linked to the recent progress in infinitely-wide neural networks?
- It would be beneficial to include a thorough sensitivity analysis for the parameters that define the topology of the neural network (like some comparison between different architectures, even with smaller neurons or layer). This is because the number of parameters used to calibrate PINNs' weights

and biases significantly impacts the overall complexity of the training process.

- In lines 619–620, the authors assess that *the proposed split PINN-based approach is a robust and straightforward tool for monitoring the initial spread of a disease.* However, I am unable to evaluate the goodness of this tool in the prediction sense, since, as I understand it, the numerical results are retrospective. They approximate the dynamics within the same period as the data used for training and have not been utilized to extrapolate future behavior. Could you provide some clarification on this aspect?

Recommendation

I recommend that the paper addresses the previous points, and then it can be accepted.
