# Supplementary material for: A Physics-Informed Neural Network approach for compartmental epidemiological models
Source: PLoS Comput Biol. 2024 Sep 5;20(9):e1012387. doi: 10.1371/journal.pcbi.1012387 (PMC11407682; doi:10.1371/journal.pcbi.1012387)
Supplement: Attachment — Submitted filename: Response letter -PLOS CB.pdf [file pcbi.1012387.s003.pdf]

Responses to the comments from Editors and Reviewers

Associate Editor

I agree with both reviewers that this work potentially represents an important contribution to the field of computational epidemiology and infectious disease modeling; however, substantial work is needed to clarify why specific modeling techniques were selected and how the results may compare to more established methods. The authors should clarify whether true out-of-sample performance was measured (not necessarily prospectively, but at least on fully held-out data). If such out-of-sample testing was not performed doing so will be essential for fully addressing the reviewer comments. I also would like the authors to revise their work to make it more accessible to a broad audience. Please pay close attention to the detailed comments from the two reviewers.

R: *We would like to thank the Associate Editor for giving us this opportunity to improve the manuscript. To address the major comments raised by the AE and the Reviewers, we modified the manuscript as follows:*

- *As requested by Reviewer 1 (see replies R1.1 and R1.2), we performed a sensitivity analysis of the errors and computational times with respect to the number of neurons and hidden layers of the PINNs. This analysis is presented in the Supporting Information S1, and better justifies the selected architectures for the NNs.*
- *We included a new section in the results (Section 2.2.2) where PINNs are applied to a forecasting framework. The Italian COVID-19 data is gradually included in the training and the NNs are used in extrapolation to produce short-term forecasts of two weeks. This analysis shows the performance of PINNs when using only a portion of the data (please see reply R2.C2) and assesses the quality of the extrapolation (please see reply R1.3).*
- *As requested by Reviewer 2 (see reply R2.C1) we also propose a comparison between the reproduction numbers obtained using the PINN approach and the renewal equation proposed by Cori et al. (AJE 2013). PINN results are overall similar to those of the renewal equation (see Supporting Information, S3). In the section 'Discussion and Conclusions', we better highlighted the benefits and limitations of the PINN approach with respect to this more established method.*
- *We finally made the manuscript more engaging by including in the introduction a simple flowchart of the joint and split approaches (Fig 1) and restructuring the Methods and Result sections following the suggestions of Reviewer 2 (see replies R2.3, R2.4, R2.C4-R2.C6).*
- *Please find in the following our replies to each detailed comment of the two Reviewers.*

Reviewer #1

The paper introduces an innovative approach to address inverse problems related to epidemiological data and utilizing PINNs. Specifically, two simple innovative modifications,

named the reduced and split approaches, are introduced and compared in a comprehensive campaign of numerical results involving both synthetic and realistic scenarios.

Strengths

1) The authors have shown a clear understanding of the existing state-of-the-art about Machine Learning techniques for solving Inverse Problems and in particular the application to the epidemiological context;

2) The proposed methods introduce little modifications adapted to the epidemiological framework to the well-established framework of PINNs.

R: *We thank the Reviewer for the positive feedback.*

Major Comments

1) I believe there is a lack of explicit justification for employing Neural Networks with large dimensions, as indicated in lines 369-373, particularly regarding their depth and width. Could this choice be somehow linked to the recent progress in infinitely-wide neural networks?

2) It would be beneficial to include a thorough sensitivity analysis for the parameters that define the topology of the neural network (like some comparison between different architectures, even with smaller neurons or layer). This is because the number of parameters used to calibrate PINNs' weights and biases significantly impacts the overall complexity of the training process.

R1.1 e R1.2: *We agree with the Reviewer that establishing the correct architecture of the Neural Networks is crucial in each PINN application. On the one hand, the number of hidden layers and neurons should be large enough to follow the temporal changes and the nonlinearities of the unknown solution. On the other hand, a low number of unknown parameters is desirable to avoid overfitting and restrict the computational costs. However, the literature does not provide clear indications on the correct architecture to adopt and the dimensions are typically a modeler's choice that depends on the particular problem. Following the Reviewer's advice, we performed a sensitivity analysis on the error of Case 1 (constant transmissivity) varying the number of layers (4 or 10) and neurons (5,25, 50, or 100). The results presented in the Supporting Information S1 (Figs SI.1-3) show that the architectures with four layers and 25 or 50 neurons provide similar accuracy with limited training times. A lower number of neurons would significantly increase the errors, while architectures with more layers or neurons would have larger training times (and sometimes larger errors). Time-variable parameters require a large number of neurons (here 100), to avoid approximation errors such as those depicted in Fig. 16. This sensitivity analysis supports our choice for the proposed architecture.*

We would like to underline that architectures with similar dimensions have been adopted in other PINN applications to epidemiological models. We report here some examples from the papers that we cited: [21] Torku et al. (2021) use 80 neurons and 5 hidden layers; [22] Malinzi et al. (2022) use 30 neurons and 3 hidden layers; [24] Schiassi et al. (2022) use 50 neurons; [25] Long et al. (2021) explores the errors associated to 2-5 layers and 16, 32, 64 neurons (and selects 3 layers, 64 neurons) to identify a time-dependent transmission; [27] Olumoiyn et al (2022) use 3 layers and 64 neurons; [28] Ning et al. (2023) use 5 layers and a variable number of neurons (between 30 and 50).

3) In lines 619–620, the authors assess that the proposed split PINN-based approach is a robust and straightforward tool for monitoring the initial spread of a disease. However, I am unable to evaluate the goodness of this tool in the prediction sense, since, as I understand it, the numerical results are retrospective. They approximate the dynamics within the same period as the data used for training and have not been utilized to extrapolate future behavior. Could you provide some clarification on this aspect?

R1.3: *In the first version of the manuscript we were mainly aiming to explore the potential of PINNs for the estimation of unknown time-varying parameters. This is an essential step to provide a good starting point for the model projections and to possibly provide a trend for the unknown parameters. To assess the performance of PINNs in exploring the future behavior of an epidemic, we added a new section of the results dedicated to short-term projections (Section 2.2.2). We considered test Case 7 and updated the model calibration every 15 days. At the end of each calibration we projected the number of infected individuals and the unknown parameters during the following 15 days as extrapolated by the calibrated neural networks. As one could expect, the projections are not accurate when few data are available or when the model is close to the peak of the epidemic. Good results have been obtained during the recession.*

Recommendation
I recommend that the paper addresses the previous points, and then it can be accepted.

R1.4: *We thank the Reviewer for her/his insightful comments. We implemented most of the Reviewer's helpful suggestions and we are confident that the manuscript strongly improved during the revision process.*

Reviewer #2

In this study, the authors apply a neural network approach informed by differential equations (Physics-Informed Neural Networks) to infer the parameters' values that regulate the dynamics of an SIR model. The results show that the approach adopted by the authors, based on a reduced-split of the training set, outperforms the methods that have been previously proposed in the literature both in terms of accuracy and computational times.

The study presents a few strengths and weaknesses at the same time, which I summarize in the following. Overall, the paper quality does not currently meet the standards to be published in PLOS Computational Biology and I think the paper requires a major revision before it can be considered for publication PCB. I am suggesting a number of changes that I hope could improve the manuscript.

Strengths:
1) The use of PINN in infectious disease modeling is promising – yet understudied – and the method proposed by the author improves on previous results, thus showing a more effective way of using PINN in this field.
2) The study considers a range of application scenarios that include real-world data, and more realistic cases than previous studies.

R: *We thank the Reviewer for the positive feedback and for her/his insightful comments. We implemented most of the Reviewer's helpful suggestions and we are confident that the manuscript strongly improved during the revision process.*

Weaknesses:

1) The main result of the study is the introduction of a novel training approach for PINNs applied to the SIR model. While I consider it interesting and useful, in terms of impact I am not sure it will be of high interest to the broad audience of PCB.

R2.1: *We acknowledge that PCB has a broad audience, however, in our opinion, the topics of the manuscript are of interest for a large part of PCB readers. A rapid research in the PCB website shows that, since December 2020, there have been thousands of papers published in PCB that used Neural Networks. More than 200 of these papers are related to epidemiological applications, meaning that there is already a large community of epidemiologists that uses and develops strategies based on NNs. PINNs are present in only 5 papers in PCB, which underlines their novelty. In particular, He et al. (PCB, 2023) used a PINN approach to study the COVID-19 outbreak in China.*

2) For the readers who are not familiar with PINNs, it will be difficult to assess the quality of the results with respect to more traditional approaches. The paper is often technical, and it is hard to compare the results against more traditional infectious disease modeling techniques.

R2.2: *We acknowledge the Reviewer's concern. We modified supporting information of the manuscript including a comparison between the Rt values estimated with PINNs and those estimated with the renewal equation of Cori et al. (AJE 2013) (SI, section S3). It is important to keep in mind that the two methods start from very different assumptions. For example, in the considered Cases 3 and 4, the PINN method estimates Rt as a temporal function of time using the whole set of data. The approach of Cori et al., instead, independently estimates Rt at each day using a Bayesian approach and considering only the data on a small window (typically collected in the past). For example, using a window of 5 days, the estimate on day d is obtained by considering Rt as constant on days d-4, …, d-1, d, which might cause a delay in the Rt changes. To make the comparison with PINNs more fair, we modified the method of Cori et al. by averaging the values of Rt on a centered window (2 days in the past and 2 in the future). The comparison shows similar results, in both Cases 3 and 4. In Case 3 Cori et al. clearly outperforms the Rt estimation of PINNs at the beginning of the epidemic. At larger times, the PINN estimation of Rt better agrees with the reference values in both Cases 3 and 4. The main advantage of Cori et al. in Case 4, where the data is subject to larger errors, is the estimation of the confidence interval which is intrinsic to this Bayesian approach. We would like to stress that the extension of PINNs to the Bayesian framework is possible (see e.g. [30] in the new ms), but it is out of the scope of this work (See R2.C7).*

3) The presentation of the results and the structure of the manuscript follows a bullet list style (Case 1, Case 2, etc.) that is not very engaging. Figures and tables repeat the same structure over and over, and they are sometimes hard to interpret.

R2.3: *One of the goals of the paper is to analyze the performance of PINNs under an increasing level of model complexity. For this reason we argue that labeling the different*

scenarios using Case 1, Case 2, etc. is useful to clearly identify in which model setting PINNs are applied. To help the reader, Table 1 summarizes the characteristics and differences of each test case. Moreover, in the new version of the ms we tried to improve the interpretation of the results by always describing in a more explicit way the features of the considered Case (without the need to necessarily consult Table 1). Finally, to make the result session more engaging, we followed the Reviewer suggestion subdividing the studied test cases between synthetic scenarios and the applications to real data. The synthetic scenarios are used to validate the proposed method knowing the values of the unknown parameters (Section 2.1, Model Validation), and comprehend Cases 1-5. Section 2.2 then describes the application to the Italian Covid pandemic (Cases 6-7) and the new model results about forecasting.

4) The paper suffers from an overly technical notation that can appear obscure to biologists, epidemiologists and infectious disease modelers.

R2.4: *The equations proposed in the manuscript are essential to describe the epidemiological model and the PINN approach. Eqs 1-2 describe the SIR model; Eqs 3-4 describe a general NN; Eqs 5-6 describe how to adapt the SIR model for the application to PINNs; Eqs 7-11 describe how to build the loss functions in the joint and split approaches. Eqs 12-16 reframe the model and the loss functions for the reduced SIR model; Eqs 17-35 explain how to adapt the procedure when including hospitalization data. Finally Eq 36 provides the model error. All these steps are needed to ensure the replicability of the proposed methods.*
As suggested by the Reviewer, we included in the introduction a new diagram that schematically describes the two PINN approaches (joint and split). We hope this could help the readers to clarify the methods proposed in the paper.

Comments and suggestions

1) It would be important for infectious disease modelers to understand if PINNs provide benefits with respect to classic methods that are used to estimate Rt in real world cases. For instance, the widely adopted Rt estimation method proposed by Cori et al. AJE 2013. Can PINNs replace these approaches to some extent? In general, it is not obvious for those who work in IDM what tasks can be accomplished by PINN with better or similar performance than commonly used methods.

R2.C1: *As described in R2.2, the new version of the manuscript now includes a comparison between the Rt estimated with PINNs and Cori et al. (see SI, section S3) At the present stage, the proposed PINN method does not have the ambition to replace more traditional approaches, such as Cori et al.. However, PINNs offer some benefits that are not directly available otherwise. For example:*

1) the simultaneous estimation of multiple parameters that change in time (e.g. Rt and sigma in Cases 5-7),

2) the inference using jointly different types of data (e.g. infected and hospitalized individuals in Cases 5-7),

3) the possibility of providing a future projection for the evaluated parameters (see new section on forecasting)

4) the possibility of training the model even if there are gaps among the data (thanks to the physical model) .

A final important consideration is that PINNs benefit from the so-called 'transfer learning' typical of Neural Networks, that is the possibility to adapt an already trained NN to new data. This has been explored, for example, in the application to forecasting (Section 2.2.2). We better underlined the benefits (and limitations) of PINN with respect to other standard methods in the Discussion.

We would also like to underline that many researchers are exploring the possibility of using NNs to tackle problems in different fields of applications and, in particular epidemiology (see R2.1). The manuscript provides a step forward in the use of PINNs in real epidemiological models.

2) On a similar note, I did not fully grasp what is the portion of the epidemic curve that is used to train the PINN in each scenario. Does the model need the full curve as training input? In this case, its use would be strongly limited in the early phase of an outbreak.

***R2.C2:** The new results about forecasting clearly reply to the Reviewer's comment (See Section 2.2.2). PINNs can be applied to any portion of the epidemic curve. In Cases 1-7 we considered the full curve because we were mainly interested in retrieving the unknown temporal changes of the parameters during the epidemic. In Section 2.2.2 PINNs are applied to temporal windows of increasing size, simulating the computation of model projections every two weeks of the pandemic. Every time that new data is considered, the Neural Networks are updated (transfer learning) so that the estimated Rt is always a continuous and differentiable function of time . It is important to note that the estimated values of the unknown parameters are similar to the reference values (those estimated using the whole set of data) also when considering few data points, underlying the robustness of the method.*

3) Choosing a fixed value for the average infectious period (5 days) seems a strong assumption. Is this kept constant in all experiments? Often, there is a large uncertainty affecting estimates of the infectious period. How sensitive are the results to changes in the infectious period value?

***R2.C3:** We agree with the Reviewer that the infectious period is typically highly uncertain. However the probability distribution of the generation times (and, thus, the mean infectious period) is kept constant in most epidemiological applications (see e.g., [2, 4, 5]).*

Please note that also in the application of the integral convolution proposed by Cori et al. the probability distribution of the generation times is typically kept constant during the simulation. For this reason the mean time for the infectious period is kept constantly and equal to 5 days in this work. In the SIR model, this is equivalent to having an exponential probability distribution of the generation times, with expected value of 0.2 d^{-1}. We adopted the same

exponential distribution for the generation times (truncated at 30 days) for the comparison with the Rt estimated with the method of Cori et al..

To make our choice clearer to the readers, we added a remark at line 165.

4) The section Methods currently describes in detail the mathematical structure of the PINN. I would suggest extending the section with a description of the numerical methods that right now appear at the beginning of the results. All the information regarding the numerical experiments (size of training, testing, error metrics, etc.) should be part of the methods.

R2.C4: *We agree with the Reviewer and moved the description of the numerical experiments in two subsections of the 'Methods': 'Simulation setup' and 'Implementation details'.*

5) A graphic description of the numerical methods could also improve the readability of the paper. A summary figure describing how the epidemiological data are used in the training phase, what is the output of the model, etc.

R2.C5: *We thank the Reviewer for this suggestion. We included the suggested diagram in the introduction (new Fig 1), so that the reader can have an immediate idea of the structure of the proposed PINN approaches (split and joint).*

6) I would recommend changing the structure of the manuscript with sections title that do not just report the names "Case 1, Case 2, Case 3…" For instance, Results could be grouped into two main parts. One considering synthetic data and the other considering COVID-19 data.

R2.C6: *We grouped the results in two groups as suggested and described the main features of the Cases when using them in the text (See R2.3).*

7) One major limitation of the PINN approach is to fit a deterministic SIR model to the data. The deterministic model leads to parameter estimates with almost exact values that are difficult to interpret when compared to traditional statistical methods used in the literature that provide a confidence interval of credible values. Is there a way to overcome this limitation by extending the range of possible outcomes of the model? I understand this is an open question that may lack a satisfying answer, but at least I would discuss this limitation more extensively at the end of the paper.

R2.C7: *We agree with the Reviewer that it is fundamental to include a quantification of the uncertainties when performing parameter inference. Unfortunately, at this stage, this is not straightforward using PINNs and ad-hoc strategies should be developed. We highlighted this limitation in the discussion (lines 658-666). We plan to further develop this aspect in our future work following, for example, the combination of PINNs with Bayesian inference proposed in [39].*
