# Supplementary material for: A Physics-Informed Neural Network approach for compartmental epidemiological models
Source: PLoS Comput Biol. 2024 Sep 5;20(9):e1012387. doi: 10.1371/journal.pcbi.1012387 (PMC11407682; doi:10.1371/journal.pcbi.1012387)
Supplement: Attachment — Submitted filename: Response Letter EPIPINN.pdf [file pcbi.1012387.s004.pdf]

Responses to the comments from Editors and Reviewers

Associate Editor

I appreciate the authors' effort on the revisions and agree with the reviewers that the manuscript has been strengthened as a result. I agree with Reviewer 3's comments that the work needs some improvements. In particular, the authors should pay careful attention to addressing concerns about whether regularization is sufficient, whether it is appropriate to use a Python package that is core to the method but is not longer being actively maintained, whether off-the-shelf deep neural networks could perform as well or better than what is proposed here, and whether the forecasting objectives are sufficiently complicated to meaningfully test the approach. I also agree that there are many places where the methodology can be more clearly specified. Lastly, the Reviewers raised concerns about whether all necessary code/data is available to reproduce the entire study. If the authors cannot provide all necessary code/data they must formally request a waive of the Journal's policies, which I cannot promise will be granted. I believe this work is interesting and could be impactful, so I hope that authors pay careful attention to the detailed comments from Reviewer 3 and address the code/data availability concerns.

***R**: We thank the Editor for giving us the opportunity to further improve our paper. We have to admit that we encountered some difficulties in implementing some of the Reviewer's suggestions because they were somehow in contrast with the comments of the first two Reviewers. In fact, during the first round of the Revision we were asked to simplify the description of the model and the technical details of the training, in order to increase the paper readability and its potential impact over a wider audience. All the Reviewers and the Associate Editor agree that the paper actually improved after the first revision. Now, Reviewer 3 asks for many technical details about the training, which clearly go in the opposite direction with respect to the first revision. For this reason, given the positive response obtained from the first round of Revision, we have decided to include most of the new technical aspects and analysis required by the third Reviewer in the supplementary material.*

We observe that in our work we focussed on the use of PINNs, whose training differs in some technical aspects from data-driven NNs and DNNs. We clarified this point whenever suitable in the main text. For what concerns the major comments raised by the Reviewer and recalled by the Associate Editor, we make the comments that follow.

*1 - **Concerns about whether the regularization is sufficient**: regularization is important in NNs to avoid overfitting on the data, which are typically affected by measuring errors. However, PINN's training is based on a loss function that includes both the data and the norm of the residuals of the governing differential equations. It is known that such norms act as a regularization term in the PINN loss function, and additional regularizations typically are not necessary (see [M. Raissi, P. Perdikaris, G.E. Karniadakis, Physics-informed neural networks: A deep learning framework for solving forward and inverse problems involving nonlinear partial differential equations, Journal of Computational Physics, Volume 378, 2019, Pages 686-707, ISSN 0021-9991, https://doi.org/10.1016/j.jcp.2018.10.045.]). For the sake of clarity, we underlined this point in the description of the loss functions used for PINN's training.*

*2 - **Concerns about whether it is appropriate to use SciANN Python package, that is no longer actively maintained**: We started working on PINNs more than three years ago thanks to a collaboration with Prof. Juanes' research group at the Massachusetts Institute of Technology. Prof. Juanes' research group is the main developer of the Python package SciANN.This is the basic reason why we adopted this software package for our analysis. The package has been regularly updated until February 2023, when we were already developing and running most of the examples presented in our paper. Unfortunately, the leader of the SciANN group recently moved to a different employment and, as a consequence, the package maintenance to the new version of TensorFlow and Python has been temporarily suspended. However, we would like to underline that the use of a specific software package, such as SciANN, is not core to the method. In our paper, we aim at presenting a novel approach that can help address some issues in the calibration and use of epidemiological models. As required by the journal policy, the code has been included to the benefit of the interested readers just as an example of how the approach we propose can be implemented. The results presented in the manuscript would only marginally change by replacing SciANN with other packages or our own routines, which could anyway encounter in the near future the same update issues with TensorFlow and Python raised by the Reviewer. Moreover, we don't see any particular scientific value in writing our own routines to implement algorithms that are already available in different packages.*

We would like to stress that the manuscript aims to propose and compare two PINN-based methodologies to tackle inverse problems with transient parameters in compartmental epidemiological models. We are interested in evaluating the differences in the accuracy and computational complexity of the Split and Joint results when using the same Python package and the same computer architecture. We do not argue that our SciANN implementation of these methodologies is the most effective one, nor that our code should be used as it is for different applications. As required by the journal, our codes are provided to assist other researchers to assess, reuse and replicate our results.

To this aim, we added the software documentation with all the information necessary to run the code. Moreover, we expanded our Github repository by including data and Python notebooks for the test cases, so that the interested researchers can assess and replicate our results. We hope that the Reviewer and the Editor would understand that the demanded big coding work would have had very low scientific advantages and no significant impact on the paper results. For this reason, we decided to keep using SciANN.

*3 - **Concerns about whether off-the-shelf deep neural networks could perform as well:** in our opinion, a comparison between PINNs and purely data-driven DNNs is not appropriate in the context of the proposed approach. The use of the epidemiological model equations is fundamental in PINNs and would be completely absent in DNNs. Using only DNNs would neglect the information on the underlying epidemiological parameters (e.g. the transmissivity rate, the reproduction number, or the ratio of hospitalization), which are fundamental to understanding the transmission dynamics. We have no doubts that DNNs would provide lower errors on the data (with possible overfitting) with respect to PINNs, however it would be impossible to estimate the unobserved model state variables and parameters as we do in our work. We better underlined these aspects in the discussion of the results.*

*4 - **Concerns about whether the forecasting objectives are sufficiently complicated:** We would like to remind that the main objective of our work is to propose a PINN-based methodology to estimate the unobserved state variables and parameters during an epidemic, i.e., solving an inverse problem, by relying on both a differential epidemiological model and the data. Using the same methodology for forecasting purposes was not one of the initial objectives of our work, and we are not sure our model is the most appropriate way to address such purposes. The analysis on the forecast capabilities of our model has been added upon request of Reviewers 1 and 2, as an example of out-of-sample performance of the model even though it was not developed for this aim. Both Reviewers agreed that the presented analyzes satisfactorily replied to their comments. For the reasons above, we don't think that complicating the forecasting objectives (how?) would be useful and scientifically sound. We are aware that the naive projections we provided can be improved, for example by integrating our PINN-based model with a forward Ordinary Differential Equation solver that uses PINN's outcome as initial condition. We are currently working on this idea, but its introduction in the present work would be in our opinion well beyond the scope of this paper. We emphasized this point in the discussion of the results.*

Reviewer #3:

The authors propose the use of PINNs to regress time-dependent epidemiological parameters from data. They propose two training strategies called joint and split and also propose a method independent of the initial condition where the initial conditions do not enter the loss function. The article has been thoroughly reviewed before and the epidemiological models used are time-tested and well established in the literature. The innovation proposed by the authors is in the design and training of the PINNs. To do this the authors use a publicly available Python module called SciANN based on Tensorflow. After the first round of reviews, the authors added an example of forecasting on held-out data. And improved several parts of the work and the draft.

However, I still find the draft lacking in some respects, and the methods used by the authors are not sufficient.

While the core idea proposed by the authors is interesting, the implementation needs more rigor and some of the conclusions drawn by the authors might be affected by this lack of rigor. The authors introduce and use concepts that are not made clear to the audience this is aimed at and the training of the NN models has not been done following best-practices from proper ablation studies. The authors have added some studies of the NN architecture in the supplementary document but this is not sufficient. The authors have not explored learning rate schedules, regularization, or dependence on activation functions.

***R**: We thank the Reviewer for the positive feedback and for the detailed analysis of the proposed PINNs. Actually, we had already explored most of the elements mentioned by the Reviewer, but had not reported the detailed results in order to simplify the presentation. We are confident that thanks to this revision we further clarified some technical aspects of the training and improved the manuscript in general. Please find in the following our replies to the detailed comments.*

Comments:

1) In lines 423-425, the authors mention calibrating the weights in the loss function using eigenvalues of the NTK and cite a reference. The authors do not put any details on what they do to calibrate the weights using an NTK. From the code, it seems like they use a built-in function in the SciANN module to do this. However, they do not show whether their errors improve by using the NTK calibration or not. I am guessing it might help with the solution of the inverse problem but not with the out-of-sample prediction.

***R1**: We thank the Reviewer for highlighting this point. The Reviewer is right: we have used the SciANN built-in function for the updating of the loss-term weights during training. The code follows what is presented in the reference paper [Sifan Wang, Xinling Yu, Paris Perdikaris, When and why PINNs fail to train: A neural tangent kernel perspective, Journal of Computational Physics, Volume 449, 2022, 110768, ISSN 0021-9991, https://doi.org/10.1016/j.jcp.2021.110768]. During training, the NTK of the neural network is computed. The NTK describes how the NN predictions change with respect to small changes in its parameters, providing a way to analyze the training dynamics of the network. The eigenvalues of the NTK give insight into the sensitivity and responsiveness of the network to parameter updates, hence the weights of the loss terms in the PINN are calibrated based on them. Specifically, this adjustment of the loss-term weights helps balance the contributions of the various loss terms (e.g., data loss, boundary condition loss, and residual loss) by scaling them according to the eigenvalues, thus ensuring efficient and stable training.*

We have added in the supplementary S1 a Table summarizing the error comparison of the training with or without the NTK calibration in Case 1. This is used to justify the choice of the NTK calibration, see also lines 440-447 in the manuscript. Our results show that the NTK estimation of the weights improves the results with respect to simply adding the different contributions of the loss function. As to the out-of-sample prediction, we have to observe that this was not one of our initial objectives. This part was added upon request of Reviewer 1 and 2, as an example of out-of-sample performance of the model even though it was not developed for this aim. We are aware that our model is probably not the most effective one for forecasting purposes and agree with the Reviewer that the NTK calibration might not help. Further analyzes on the forecast predictions are currently ongoing and require more substantial modifications to our approach, including the integration of forward ODE solvers. For this reason, we prefer not focussing on the role of NTK calibration for the out-of-sample prediction.

2) From the supplementary material on the NN ablation study, it is not clear why the authors get larger errors for models of higher complexity. The authors seem to train the models on a fixed number of epochs. If they do so, more complex models are bound to overfit as the authors do not mention using any explicit regularization of the weights of the NN. It is straightforward to understand that NNs of greater complexity will be prone to overfitting if no implicit or explicit regularization is used. I would suggest the authors use early stopping as an implicit regularizer and/or add regularization to the loss function and check how that affects the NN training. This can potentially reduce training times too.

***R2**: Regularization is a technique used to prevent overfitting by adding a penalty term to the loss function. The residual term introduced in the PINN loss function acts in a similar way by penalizing deviations from the physical laws represented by the ODEs (see [M. Raissi, P.*

Perdikaris, G.E. Karniadakis, Physics-informed neural networks: A deep learning framework for solving forward and inverse problems involving nonlinear partial differential equations, Journal of Computational Physics, Volume 378, 2019, Pages 686-707, ISSN 0021-9991, https://doi.org/10.1016/j.jcp.2018.10.045.]). By minimizing the residuals of the ODEs, the NN is prevented from fitting the training data too closely, capturing noise, and leading to overfitting. Moreover, the residual term encourages the network to find solutions that are consistent with the physical laws, thus reducing overfitting and improving generalization, as a regularization mechanism usually does. For this reason, the introduction of additional regularization terms is not necessary with PINNs, and sometimes not even advisable because they could result in a more complex and harder to solve multi-objective optimization problem. We underlined this consideration at lines 252-255 of the new version of the ms.

Getting larger errors for models with higher complexity is also a known issue with PINNs, see for instance [L. Lu, X. Meng, Z. Mao, G. E. Karniadakis DeepXDE: A Deep Learning Library for Solving Differential Equations, SIAM Review 2021 63:1, 208-228]. This is due to the fact that the PINN total error can be decomposed into three main components:

- *the approximation error, which measures how closely the best possible function in the NN space can approximate the true solution of the governing ODEs;*
- *the generalization error, which is the difference between the NN trained on the training set and the best possible function in the function space;*
- *the optimization error, which is the difference between the trained NN and the NN at the global minimum of the loss function.*

These three components are inseparable and affected by different factors. The generalization error arises from the NN performance on unseen data and it is influenced by the number and location of points for the computation of the residual in the training set, while the optimization error depends on the complexity of the loss function and the quality of the optimization process. In general, NNs of higher complexity have smaller approximation errors, but higher generalization and optimization errors because the minimization of a more complex loss function is usually less effective. Therefore, the PINN training differs from that of traditional NNs, typically leading to the behavior shown in the Supplementary Material with a minimum global error at a trade-off architecture. It is also worth noting that the training is performed on a noisy data set - whose size is kept fixed - while the errors are later computed with respect to the true solution.

For all these reasons, it is not unusual with PINNs that NNs with higher complexity are less effective, with no particular relationship with the number of epochs. We added these considerations in the Supplementary Material commenting on the error diagrams.

3) The actual time taken by the NNs to train is not meaningful as the authors do not specify the computer architecture they use. The code provided by the authors is not well documented and is a single Python notebook that does not allow one to reproduce all the results. It looks more like a sample. Moreover, the authors use the package SciANN which is limited to Python <= v3.10 and works only with Tensorflow < v2.11. The training times and accuracy of the NN can be highly dependent on the Tensorflow version. The authors do not specify which version of SciANN/Tensorflow they use. If the authors want their work to be useful in the future they should write a code that can be used without much trouble in the

future. The authors could try implementing a PINN in Tensorflow or Pytorch. I know the objective of the paper is about training strategies, but many of the conclusions the authors come to can be dependent on the fact that they use a deprecated framework for their NNs (SciANN is no longer maintained as of 02/2023).

***R3**: We started working on PINNs more than three years ago thanks to a collaboration with Prof. Juanes' group in MIT, who developed the Python package SciANN.This is the main reason why we adopted this package for our analysis. The package has been regularly updated up to February 2023. Unfortunately the development group leader recently moved to another employment and this is why the maintenance of the package to the new version of TensorFlow and Python has been temporarily suspended. Having said this, we expect that the results presented in the manuscript are only marginally influenced by the choice of the package. As required by the journal, we included our code to assist other researchers to assess and replicate the results, not as the core of our work. We are mainly interested in developing a novel approach, rather than providing a code to be used as a black-box.*

We would like to stress that the manuscript aims to propose and compare two PINN-based methodologies to tackle inverse problems with transient parameters in compartmental epidemiological models. We are interested in analyzing the differences in the accuracy and computational complexity of the Split and Joint results, and we believe that such an outcome is only marginally impacted by the choice of the software package and computer architecture, provided that the same options are adopted for both the Split and the Joint methods. We do not argue that our implementation is the most effective one, nor that our code should be used as it is for different applications, and we don't see a specific scientific value in coding something that is already available from an existing package.

For these reasons, we decided to keep using SciANN and improve the quality of the documentation in order to help the interested researcher in using our code for both reproducibility purposes and as a potential guideline to develop other tools and applications. We added the documentation with all the information necessary to run the code. Moreover, we expanded our Github repository by including a Python notebook for each test case reported in the paper. We specified in the manuscript the computer architecture (A machine with two Intel(R) Xeon(R) CPU E5-2680 v2 @ 2.80GHz and 256GB of RAM, see line 445) used to obtain the results. Of course, changing the computer architecture one might expect different computational times, but the relative differences between the Split and Joint approaches should be maintained, as it has been confirmed also by the computational tests provided by the Reviewer.

4) In SI S3, the authors show that using the renewal equation approach of Cori et al. leads to comparable or better results than using PINNs. Given the complexity of the approach proposed by the authors, what is the real advantage of using their method other than just applying machine learning to this problem?

***R4:** We believe that PINNs can offer some benefits that are not directly available by the application of the renewal equation. For example:*

- the simultaneous estimation of multiple parameters that change in time (e.g. Rt and sigma in Cases 5-7),

- the inference using jointly different types of data (e.g. infected and hospitalized individuals in Cases 5-7),

- the possibility of providing a future projection for the evaluated parameters (see the section on forecasting)

- the possibility of training the model even if there are gaps or large errors or uncertainties on the quality of the data (thanks to the use of the differential model).

We would also like to underline that many researchers are exploring the possibility of using NNs to tackle problems in different fields of applications and, in particular epidemiology. The manuscript provides a step forward in the use of PINNs in real epidemiological models. This has been underlined in the Introduction and better emphasized in the concluding section.

5) In their response, the authors emphasize: “A final important consideration is that PINNs benefit from the so-called ‘transfer learning’ typical of Neural Networks, that is the possibility to adapt an already trained NN to new data. This has been explored, for example, in the application to forecasting (Section 2.2.2).” The authors are not clear about what they call “transfer learning”. It's a very loaded term and can mean several different things. The authors do not specify their strategy for transfer learning. Do they freeze the weights and add layers to the NN or do they continue the training with the same architecture as before just adding more data? If it is the latter, then it is not really transfer learning as data from the same domain and modality is being used for further training the same architecture without any fixed weights. I suspect that without using their version of “transfer learning” the NNs would do equally well leading to a lack of reason for using this transfer learning method. Please refer to A Comprehensive Survey on Transfer Learning by Zhuang et al. for more details. I would strongly recommend the authors to not incorrectly refer to their PINNS using transfer learning.

R5*: We thank the Reviewer for the clarification on the use of the term ‘transfer learning’. As suggested, we replaced this term in the manuscript by simply stating that we update the PINNs with the new available data. We clarified how this is done in lines 596-598.*

6) The authors claim: “As one could expect, the projections are not accurate when few data are available or when the model is close to the peak of the epidemic (Figs. 12a and 12b). Good outcomes are achieved during the recession (Figs. 12c and 12f).” How is the approach proposed in this paper better than that proposed in Emergence of universality in the transmission dynamics of COVID-19 by Paul et al. or any other models that perform significantly better during recession than during the growth of the epidemic? If not, then the PINN approach proposed in this work is, at best, a descriptive approach and not a predictive approach which makes the use of PINNs in this case a bit questionable.

R6*: We would like to underline that the model we propose was developed with the main aim of solving an inverse problem, i.e., estimating the values of unobserved states or model parameters. The analysis about the forecasting was added upon the request by Reviewer 1 and 2 in order to verify the performance of the proposed method “as it is” on out-of-sample data. We are aware that our method was not developed with the objective of providing accurate forecast predictions, nevertheless it appears to behave in a reasonable way and quite similarly to many other epidemiological models. The performance of our PINN-based*

model for forecasting purposes could be improved by integrating it with a forward ODE solver that uses the PINN outcome as initial condition, but this development (currently under investigation) is not trivial and beyond the scope of the present work. Also considering other sets of epidemiological data as in Paul et al might help the development of universal laws and thus more flexible models, but we believe this is outside the scope of the manuscript as well. We clarified this point in the discussion of the results (lines 690-693).

7) I do not understand why the authors create their own accuracy metric (eq. 36) rather than using standardized ones like R^2 which has a very clear interpretation.

R7*: The metric we adopted to evaluate the performance of the model is the well-known relative error in 2-norm, which is very standardly used to measure the accuracy of the solution of differential equations. The R^2 metric measures how well the model results approximate the data with respect to the mean of the data, but this would not provide a full view of the outcome of our PINN-based model. R^2 would be meaningful for the predictions in Case 7, however this part was not core to our method, as already underlined in reply R6, and we prefer to avoid the introduction of new metrics only for this case. We would like also to stress that the error metrics are here mainly adopted to compare the relative accuracy between the Joint and Split approaches, not to provide absolute results.*

8) The simulated scenarios are very simple and I suspect even a DNN can be used to find the solution. The authors do not demonstrate that their PINN outperforms a simple DNN implementation.

R8: *In our opinion, a comparison between PINNs and purely data-driven DNNs is not appropriate within the context of our work. The use of the epidemiological model equations is fundamental in PINNs and would not be present in DNNs. Using only DNNs would neglect the information on the underlying epidemiological parameters (e.g. the transmissivity rate, the reproduction number, or the ratio of hospitalization) which are fundamental to understanding the transmission dynamics. No doubt DNNs can provide lower errors on the data (with possible overfitting) with respect to PINN, however it would be impossible to estimate the unobserved model state variables and parameters. We better underlined these aspects in the discussion of the results (lines 707-716).*

9) I do not see a comparison between split and reduced-split nor where the reduced-split method significantly outperforms the split method. The code does not seem to have any examples of the two separately.

R9: *Actually, we did the comparison between the full SIR model and the reduced one, but it was not presented in the manuscript to limit the number of test cases. Indeed, Reviewers 1 and 2 had observed that the paper already contained too many cases and for this reason was not "engaging" for a wide audience. For this reason, we added such a comparison in the Supporting Information (see new section S2 Appendix. SIR model with large data errors.), specifically the results obtained with the full SIR model in Case 4 (synthetic transmission rate with large data errors) (see also lines 542-545).*

10) For the case of split training, it seems like the SciANN module is using a reduce-on-plateau learning rate schedule where the learning rate is reduced by a factor of 2 on plateau of the validation loss. Hence, the authors' statement in section 1.6 that they use a

learning rate of 0.001 in all cases is incorrect. Secondly, it is well known that learning rate schedules significantly improve training. I'd encourage the authors to explore the use of learning rate schedules in training the PINNs.

R10: *We thank the Reviewer for the suggestion. We specified in the manuscript (lines 430-431) that we used a reduce-on-plateau learning rate schedule. We know that learning rate schedules generally help the training and we have already used them in other applications (e.g., [C. Millevoi, N. Spiezia, M. Ferronato, On Physics-Informed Neural Networks Architecture for Coupled Hydro-Poromechanical Problems. Available at SSRN: https://ssrn.com/abstract=4074416 or* http://dx.doi.org/10.2139/ssrn.4074416*]). However, in this case the gain was not significant.*

11) Using Tensorflow 2.11 and Keras 2.11 I get an order of magnitude smaller errors that the authors get with the code they have shared in their repository. Since the authors did not specify the versions of the modules they used, it is impossible for me to say whether the improvement is due to the module version or the initialization of random numbers (unlikely) that have not been seeded in the provided notebook.

R11*: We thank the reviewer for underlying this point. We now better specify the version of Tensorflow used to obtain our results in both the manuscript (line 446) and the 'requirements.txt' file in Github. Another possible reason for the different results obtained by the Reviewer is that the errors presented in the manuscript are computed as the average error over 10 different trainings of PINNs. We would like to further stress that the main focus of the paper is not obtaining the minimum error, but comparing the accuracy and computational efficiency of the split and join approaches in the different test cases.*

12) The authors have provided no insight as to why the split method works better than the join. In a real-world scenario, it might be imaginable that the infection rate is affected by real-world uncertainties like testing strategies etc., in which case a data-driven approach to this estimating the infection rate might work better. However, for simulated data, this is not the case. So, some insights into why split training works better would be useful.

R12: *We thank the Reviewer for raising this point, which gives us the opportunity to better underline a potential advantage of the use of PINNs. In fact, the PINN-based approach can work better than a purely data-driven approach especially when the data are subject to large uncertainties, because the residual on the model equations acts as a regularization term and helps drive the solution to a physically consistent result even when the quality of data is limited. As to the insight as to why the split method outperforms the joint one, this is basically due to the structure of the loss functions. Splitting the training implies minimizing two loss functions which are simpler than the multi-objective optimization required by the joint approach. Since the final PINN error depends on the structure of both the NN and the loss function, as already clarified in reply R2, this generally leads to shorter training times and higher accuracies. We added these considerations in lines (669-672).*

The fork of the repository run with TensorFlow 2.11.0 can be found here where the discrepancy in error estimates can be verified (the requirements file specifies the module versions): https://github.com/anonymousacademicc/EpiPINN

<u>Have the authors made all data and (if applicable) computational code underlying the findings in their manuscript fully available?</u>

The PLOS Data policy requires authors to make all data and code underlying the findings described in their manuscript fully available without restriction, with rare exception (please refer to the Data Availability Statement in the manuscript PDF file). The data and code should be provided as part of the manuscript or its supporting information, or deposited to a public repository. For example, in addition to summary statistics, the data points behind means, medians and variance measures should be available. If there are restrictions on publicly sharing data or code —e.g. participant privacy or use of data from a third party—those must be specified.

Reviewer #1: None

Reviewer #2: Yes

Reviewer #3: **No:** The authors have only provided sample code and not all the code necessary to reproduce their work. They have specified a link to the data but have not provided code for the ETL of the data. The link they provide simply leads to a landing page: https://www.epicentro.iss.it/en/coronavirus/. With the information and the code that they provide it is impossible to reproduce their results more so because they have not specified the version of the python modules used. Moreover, they use a python module that is no longer supported by the developers and is deprecated. The module depends on interpreters and modules that will be deprecated in the near future limiting the utility of the computational method that they develop and inhibiting the reproducibility of their work.
